# Beyond prediction error: 25 years of modeling the associations formed in the insect mushroom body

**DOI:** 10.1101/lm.053824.123

**Published:** 2024-05

**Authors:** Barbara Webb

**Affiliations:** School of Informatics, University of Edinburgh, Edinburgh EH8 9AB, United Kingdom

## Abstract

The insect mushroom body has gained increasing attention as a system in which the computational basis of neural learning circuits can be unraveled. We now understand in detail the key locations in this circuit where synaptic associations are formed between sensory patterns and values leading to actions. However, the actual learning rule (or rules) implemented by neural activity and leading to synaptic change is still an open question. Here, I survey the diversity of answers that have been offered in computational models of this system over the past decades, including the recurring assumption—in line with top-down theories of associative learning—that the core function is to reduce prediction error. However, I will argue, a more bottom-up approach may ultimately reveal a richer algorithmic capacity in this still enigmatic brain neuropil.

Understanding how brains implement learning is crucial to explain the flexibility of animal behavior. The insect mushroom body (MB) has attracted increasing research attention as evidence accumulates that modulation of connections in this circuit forms a fundamental basis of learned associations (see papers in this issue). But learned associations between what? In Webb (2013), I discussed the plurality of interpretations possible when an animal is observed to alter its behavior to a stimulus as a result of pairing that stimulus with innately rewarding or punishing events (e.g., a fly showing increased attraction or aversion to a previously neutral odor depending on whether it has been paired with sugar or electric shock). Does this behavior mean there is now a neural connection from odor sensing to sugar sensing, resulting in the same behavior to both? Or from odor sensing directly to the behavior caused by sugar? As outlined in a crucial paper by Rescorla (1988), neither of these putative associations captures the observed phenomenon, as the behavior to the odor (typically, reorientation in a concentration gradient) is not identical to that displayed to sugar or shock (respectively, ingestion or freezing). Alternatively, is the association formed between odor and a particular behavior toward odor (e.g., either positive or negative chemotaxis, as appropriate to whether the odor predicts reward or punishment)? Or more abstractly, does the odor become associated with a positive or negative predicted value that can flexibly influence behavior according to the current needs and situation of the animal? The latter seems to be emerging as the dominant interpretation.

Although there may be reasons (indeed, some are already raised in Rescorla 1988) to consider the associated prediction as richer than a single dimension of value, for the purposes of the following discussion, I will assume a key question to answer in any model of the MB is the following: How does MB circuitry support the establishment of neural connections that encode the association of a sensory pattern to its predicted value, based on the animal's experience of innately valuable events being contingent on the occurrence of the sensory pattern? Not surprisingly, formulating the question in this way leads to an obvious theoretical answer, most commonly referred to in the form given by Rescorla and Wagner (1972): An animal will alter its prediction of the value of a sensory pattern proportionally to the error it perceives between the predicted value and the actual value experienced. This assumption remains the most widely accepted and influential account of associative learning as well as lying at the heart of modern machine learning.

Several key insights and a range of behavioral results follow from this prediction error (PE) formulation. First, contingency rather than simple contiguity is the key to the association: If reinforcement co-occurs with a stimulus equally often but in a random rather than a consistent way, the association will not be formed (or, more precisely, this will result in increases and decreases in associative strength that cancel out). Second, if there is a contingent relationship, the associative strength will initially increase rapidly but the rate of increase will slow as the prediction asymptotically approaches the value of the reinforcer. If the reinforcer is subsequently increased in value, the association strength should undergo a further increase. Similarly, if the reinforcer is omitted, there will be a negative error that should weaken the strength of the association. Note that in this formulation, the occurrence of a reinforcer without the stimulus should not alter the value associated with that stimulus.

An original motivation for this influential account of the associative learning process was that it explained the phenomenon of blocking (Kamin 1969). If one sensory pattern already predicts a reward, then the presentation of a second stimulus along with the first, followed by a reward, should not produce any positive association of value to the second stimulus, as there is no error in the prediction. One reason for some skepticism over the applicability of PE to insect learning has been the lack of robust demonstration of blocking in insects. Smith and Cobey (1994) reported blocking in bees for combined odor presentations, but the phenomenon was not clearly reproduced under careful controls (Gerber and Smith 1998; Gerber and Ullrich 1999); similar unimodal experiments in flies failed to show a blocking phenomenon (Young et al. 2011), but cross-modal blocking has been shown in crickets (Terao et al. 2015; Terao and Mizunami 2017). However, it is important to note that whether a PE process should produce blocking in any particular circumstance depends on assumptions about how the simultaneous presentation of two stimuli together is actually experienced or encoded by the animal. For example, presenting a combination of two odors might be experienced as a novel new odor or at least modify the perception of the previously learned component sufficiently to modulate the prediction.

The plausibility of PE as a fundamental insight into biological learning was famously strengthened by the observation that dopaminergic neurons (DANs) in the monkey midbrain appeared to have activity patterns that matched the PE during learning tasks (Schultz et al. 1993). Specifically, it was observed that their response to the occurrence of reward was reduced as the animal learned that reward was predicted by a sensory cue, and indeed the onset of activity was now associated with the occurrence of the predictive cue. Particularly striking was that the omission of the reward after the cue resulted in a reduction of dopaminergic activity below baseline, matching the “negative prediction error” of the theoretical account.

The following survey of computational models of learning in the MB focuses on the ebb and flow of PE as a guiding principle in interpreting the circuit function. For the most part, I will discuss the “insect MB” generically, rather than distinguish between species—although there may be important differences, these are largely unaddressed in computational modeling to date. The basic features common to all models (see Fig. 1, top) are the assumption that sensory inputs are relayed to the calyx of the MB and are encoded by a pattern of activity in the Kenyon cells (KCs); that KC outputs converge, in the lobes, on a small number of mushroom body output neurons (MBONs) that will ultimately affect behavior; and that the synapses between KCs and MBONs are plastic. Most models additionally assume there is further MB input, influencing the plasticity, from neurons signaling reinforcement, usually in the form of dopamine (DANs). My aim here is not to examine this neural architecture in detail (see an excellent recent review in Modi et al. 2020, or other papers in this issue) but rather focus specifically on the hypothesized interaction of inputs, KCs, MBONs, DANs, and outputs that have been proposed and evaluated in computational models to date (for clarity, I will use the KC/DAN/MBON terminology throughout even though different terms might have been used originally in the papers discussed).

**Figure 1.**
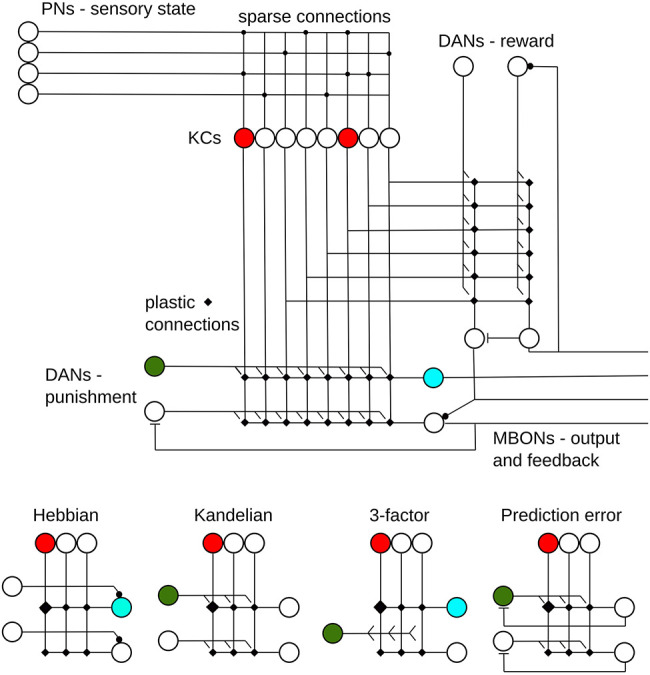
Key features of the mushroom body (MB) and proposed learning rules. (*Top*) Sensory input is carried by projection neurons (PNs) which make sparse connections to MB Kenyon cells (KCs). KC axons bifurcate and connect to a small number of MB output neurons (MBONs). Reward and punishment signals are carried by dopaminergic neurons (DANs), which influence the plastic KC–MBON connections. MBONs also connect (selectively) to each other and in feedback loops (direct and indirect) to DANs; these connections can be excitatory or inhibitory. Additional features not illustrated include neurons conveying global inhibitory feedback from the lobes to the calyx, connections between KCs, and direct connections from KCs to DANs and from DANs to MBONs. (*Bottom*) Four broad classes of learning mechanisms have been proposed in the models discussed in the text (see also Table 1): Hebbian, in which simultaneous KC and MBON activation strengthens the synapse between them (this might assume that the MBON's initial activation is caused by direct input from a DAN); Kandelian, in which DAN activation releases a neuromodulator that strengthens the active KC synapses onto MBONs; three-factor, in which Hebbian learning is gated by DAN activation; and prediction error, in which Kandelian learning is modulated by negative feedback from MBON to DAN (note that if coincidence of KC–DAN activity is assumed to depress the synapse, then “negative feedback” would be implemented by an excitatory connection from MBON to DAN).

In particular, I will focus on the different learning rules that have been implemented and their contrasting assumptions on how the relative activity of KC, MBON, and DAN should affect synaptic changes between them (Fig. 1, bottom). Consequently, I will not discuss models that focus primarily on how sensory stimuli are encoded by KCs, or on other salient features of this circuit, which may nevertheless be important in ultimately understanding how and what it computes. The survey is intended to be representative, rather than comprehensive; and provides largely qualitative rather than strict algorithmic descriptions (see also Table 1). Nevertheless, it is hoped it will provide clear insight into some key computational questions that remain unresolved, and motivate new approaches (both in modeling and neurobiology) to resolve them.

**Table 1.**
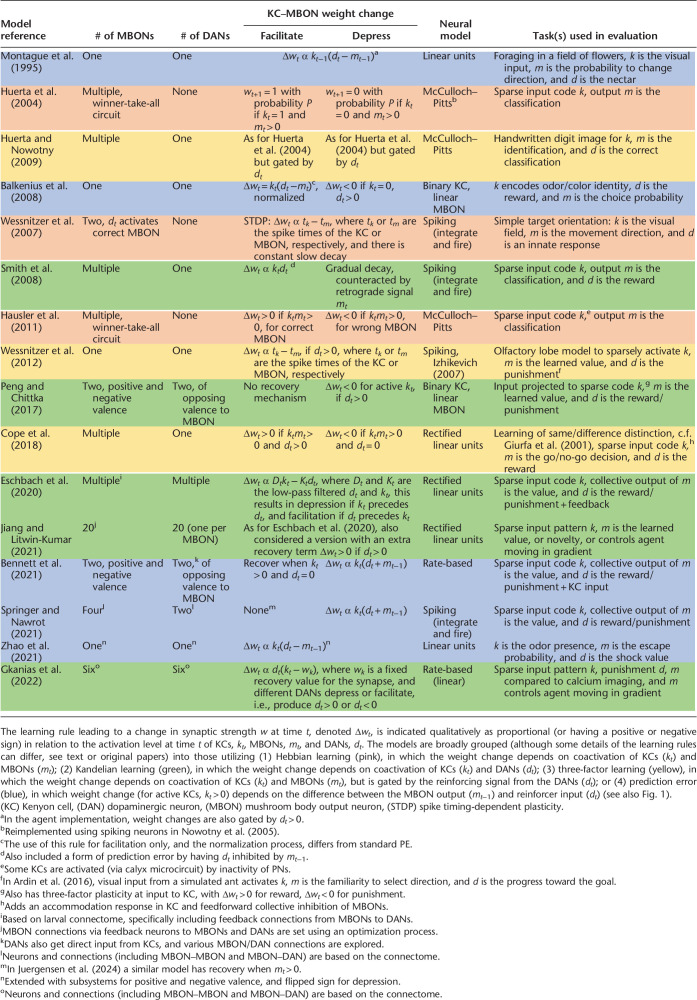
Comparison of a subset of mushroom body (MB) models discussed in the text

## Early models

The first computational model claiming direct inspiration from neural data from the MB was presented by Montague et al. (1995). Specifically, it drew on reports from Hammer (1993) and Hammer and Menzel (1995) of the response properties of the VUMmx1 neuron in the bee. This octopaminergic neuron located in the suboesophageal ganglion and projecting to the antennal lobe, lateral protocerebrum, and MB calyx shows a strong and prolonged response to sucrose; depolarization of this neuron can be substituted for sucrose in odor conditioning experiments; and the response of VUMmx1 to an odor is differentially increased after pairing of that odor with sucrose. However, although Hammer and Menzel (1995) and Hammer (1997) both suggest that MB output could feed back to VUMmx1 to reduce its response to predicted reward, such a reduction appears to be a hypothesis rather than a quantified observation (one example trace, not clearly linked to a published source, is presented in a later review paper [Menzel 2001]).

In actual implementation, the model in Montague et al. (1995) does not provide an explicit parallel to known structures in the MB other than assuming the MB provides a convergence zone for sensory input (in this case visual) and reinforcement signals (based on taste or ingestion of nectar), and that its output should bias orienting actions (here a decision to continue straight or randomly reorient). For example, it does not effectively distinguish whether synaptic change could be taking place in the calyx (input to KCs, the target of the VUMmx1) or lobes (output of KCs onto MBONs, considered the key locus for learning in most subsequent models). Indeed, with respect to modeling learning, it is essentially equivalent to several earlier simulations, in which the Rescorla–Wagner rule was used to match data from learning paradigms in bees (Couvillon and Bitterman 1985; Greggers and Menzel 1993). In these earlier studies, it was noted that some modifications to the rule were needed to account for the data, including differential learning rate parameters for positive and negative PEs, a nonlinear function from predicted value to behavioral choice, and the inclusion of both short- and long-term memory factors.

Nevertheless, the model presented in Montague et al. (1995) raises some interesting issues that are still relevant to more recent models. One is the question of how to interpret the output of a neural model. Many subsequent models assume that the activity of simulated MBONs can be directly compared to the learning index produced in experimental paradigms involving multiple directional choices over time by multiple individual animals (e.g., Wessnitzer et al. 2012; Peng and Chittka 2017; Bennett et al. 2021). Others argue that the circuit needs to be embedded in a behavioral simulation to really understand how it affects observed outcomes in animal experiments (e.g., Helgadottir et al. 2013; Jiang and Litwin-Kumar 2021; Gkanias et al. 2022). In Montague et al. (1995), they suggest that the simulated bee provides useful insight into how the “structure of the environment played an important role in shaping [its] behavioral decisions.”

A second issue is whether the output of the MB circuit is considered as a monolithic signal (using either just one MBON, or assuming the balance of activity across MBONs represents value or causes behavior) or as supporting multiple, potentially independent associations (e.g., one MBON encoding the association of odors with reward, and another MBON of odors with punishment, or even more fine-scale category distinctions for individual MBONs). In Montague et al. (1995), there is a single output, which effectively sidesteps the issue of how to make the alteration of KC activity selective in its effect on different MBONs. This is relevant to the issue of calyx versus lobe as the site of learning. Although there may indeed be adaptive processes in the calyx that change the responsiveness of KCs to rewarded or punished odors, these effects would be relayed equivalently to all MBONs to which those KCs connect.

A final issue is a terminological one. The learning rule in Montague et al. (1995) is described as an implementation of “predictive Hebbian learning,” following the approach in Sutton and Barto (1981). Hebb's rule specifies that a change in the strength (or “weight”) of a synaptic connection between two neurons depends on the correlated activity of the respective pre- and postsynaptic neurons (and no other factor: “neurons that fire together wire together”). As presented, the rule in Montague et al. (1995) is Hebbian in the sense that the weights between the sensory neurons and the output neuron P (which also receives the reward as input) are altered based on their joint activity; however (to obtain a PE signal), P's activity depends not just on its current inputs (reward plus weighted sensory inputs) but on the difference of this value from the “prediction” made by the weighted sensory inputs alone on the previous time step. This inherently implies some additional factor is involved to enable the appropriate comparison to occur. That is, if neurons are modeled as single compartments (as is the case for nearly all models discussed below) such that their output activity is a function of the sum of their weighted inputs, then a simple correlation between the activity of two connected neurons cannot provide sufficient information to derive a PE signal to guide a change in synaptic strength between them. On the other hand, it is possible in more complex neural models to devise mechanisms of interaction of dendritic activity traces, or backpropagating spikes, etc. to create such a signal (e.g., Mikulasch et al. 2023). However, as will be discussed further below, the most recent “prediction error” encoding models of the MB do not take this form, but instead suggest an external circuit motif (in practice, involving the DAN, although it is not essential that this signal is neuromodulatory) to perform the comparison of predicted to expected reward. As such, these models are (for the most part) no longer Hebbian, because the activity of the postsynaptic MBON does not (directly) enter into the learning rule that changes the weight of its input from a specific presynaptic KC.

## Hebbian learning

Perhaps surprisingly, the subsequent generation of computational MB models moved away from a PE framework and instead focused on the MB as a classification system, explicitly addressing the selective strengthening of KC inputs to particular MBONs. Hebbian association was taken to be the key mechanism, in a particular form: It was assumed that the coactivation of a sparse set of KCs with a specific MBON would result in a strengthening of the connection between them. This approach was motivated at least in part from the demonstration that in the locust MB, spike timing–dependent plasticity (STDP) could be observed between KCs and MBONs (Cassenaer and Laurent 2007). For example, in Wessnitzer et al. (2007), simple visual pattern learning is explored in a spiking network with sparsely activated KCs altering, through STDP, the weight of their connection onto two MBONs. The relevant MBON was caused to be spiking in the relevant temporal relation to the KCs by being activated directly by an innate reflex pathway to avoid punishment. Note that in contrast to PE, a Hebbian learning rule provides no implicit asymptote for the synapse strength. As a consequence, this model (and many subsequent ones) assumed that synaptic strength eventually saturates, beyond which no further change occurs (alternatively, if using an “anti-Hebbian” rule, in which synapse strength decreases when neurons fire together, there is a natural limit of zero effectiveness). Synapses in this model could be weakened either through uncorrelated KC–MBON firing (the negative part of STDP) or a slow constant decay producing a “forgetting” process.

In fact, an earlier MB model from Huerta et al. (2004) embodied a similar learning principle in a more abstracted form. Using McCulloch–Pitts neurons (i.e., units that compare the sum of their inputs to a threshold and output a 1 or 0) and binary KC–MBON synapses, it is also assumed that input patterns are projected as a random sparse encoding in KCs, and a Hebbian association to MBONs is formed. In this case, the rule is that KC–MBON synapses are switched from 0 to 1 with a certain probability when a KC and MBON are simultaneously active, and to zero with a different probability when the MBON is active without the KC. A process of competition between MBONs (lateral inhibition creating a winner-take-all circuit) leads to one particular MBON becoming associated with each class of similar input patterns. It is demonstrated that this is sufficient for the classification of random patterns with different degrees of overlap and argued that the circuit is functionally analogous to the computation in a support vector machine (Burges 1998). This model architecture was implemented in a spiking network form in Nowotny et al. (2005) and used to predict an upper limit for the number of odor classes the MB could discriminate relative to the number and connectivity of neurons. A variant on the (nonspiking) model (Muezzinoglu et al. 2008) used a supervised learning approach in which the “correct” MBON is directly activated, similar to Wessnitzer et al. (2007) above.

In Hausler et al. (2011), synapses for active KCs are altered only for the strongest responding MBON, with the sign of the weight change modulated in a supervised fashion (i.e., weights of active KCs increased if this was the “correct” MBON output, and decreased if it was “incorrect”). In this investigation, it was shown to be necessary to also include calyx microcircuits such that some KCs would signal when a PN was not active to be able to separate stimulus classes that were not separable on the identity diagonal.

## Kandelian learning

An alternative “Kandelian” learning rule was proposed in the MB model presented in Smith et al. (2008), following insights into synaptic plasticity mechanisms underlying classical conditioning in *Aplysia* (Hawkins et al. 1983; Carew et al. 1984). This was motivated by the increasing body of evidence (beautifully synthesized in Heisenberg 2003) that memory formation in the MB was crucially dependent on the coincidence of KC activity and neuromodulator (specifically dopamine) release and could still occur in the absence of MBON activation (Dubnau et al. 2001; McGuire et al. 2001; Schwaerzel et al. 2003). The model in Smith et al. (2008) used a single DAN that released a neuromodulator in response to a reward and produced short-term facilitation of the connection of any active KCs to MBONs, irrespective of MBON activity. However, long-term consolidation in this model did depend on MBON activity: It was assumed a retrograde signal would change the baseline strength (i.e., the value toward which it decays following short-term facilitation) of any KC synapse with currently facilitated connections to that MBON. This allows, over time, for selective association of different patterns to different MBONs. In fact, this mechanism could set up a positive feedback loop that could further strengthen selected synapses even in the absence of the neuromodulator. This model also included an inhibitory feedback from MBONs to the DAN, effectively forming a PE circuit that would limit further learning for patterns already associated with an MBON output.

Another non-Hebbian learning rule was explored in a more abstracted model of conditioning in hawkmoths (Balkenius et al. 2008). Here, it was assumed that when a reward occurs, active KCs will have synapses strengthened (toward an asymptote set by reward strength) and also that inactive KCs will simultaneously have synapses weakened (by a constant amount) such that the MBON forms a “template” of the rewarded pattern. Note this model did not address the issue of selecting a specific MBON to allow different templates to form.

Helgadottir et al. (2013) describe a “reward-modulated Hebbian plasticity,” which multiplies a reward signal with an eligibility trace following spikes in KCs to alter their synaptic weight onto MBONs. The spiking network is implemented on a robot that learns to approach a color input associated with a reward. It is not fully clear if the implemented rule is in fact meant to represent a (non-Hebbian) Kandelian mechanism (i.e., the release of neurotransmitter from a DAN modulating active KC synapses independently of MBON activity) or whether it is assumed the reward signal is activating the MBON—hence, a Hebbian rule similar to that described above for Wessnitzer et al. (2007). This confusion—using “Hebbian” to describe a change in KC–MBON synapses caused by the simultaneous firing of DAN and KC, independently of MBON activity—remains a common one in more recent papers and can obscure important differences between models.

It is relevant to note that in the models discussed so far (and several that follow), it was assumed that associative learning in the MB principally involves a facilitation of KC–MBON synapses from initially weak connections. However, direct evidence was building that paired activation of DANs and KCs (or more specifically, DAN activation following KC activation) produces synaptic depression, such that later presentation of the same stimulus, activating the same KCs, will produce a weaker output from the corresponding MBONs (Séjourné et al. 2011; Cohn et al. 2015). Later models that mimic this effect often describe it as an “anti-Hebbian” learning rule but as above, this is misleading if the activity of the postsynaptic MBON has no direct influence on the synaptic change. Considered in purely computational terms, potentiation versus depression of the synapse does not make a critical difference to the models discussed here, which are sufficiently abstract that a simple change in the sign of the learning rule, and some corresponding adjustments in positive or negative effects on behavior or feedback, can produce equivalent phenomenological results.

## Three-factor models

Although described as Hebbian, the learning rule used in Montague et al. (1995) in practice included an additional term (not shown in the equations) that gated any synaptic change to occur only when the simulated bee was in contact with a flower. A gating signal for the occurrence of learning was later (Huerta and Nowotny 2009) added to the Hebbian MB model described in Huerta et al. (2004) when it was used to perform the classification task of handwritten digit recognition (a standard benchmark for pattern classification). That is, in Huerta and Nowotny (2009), a particular KC–MBON connection is strengthened, when both are active, only if the “correct” MBON has been activated, with this modulation assumed to be carried by a reinforcing signal. In Bazhenov et al. (2013), using a similar architecture to Huerta et al. (2004) and Huerta and Nowotny (2009), unsupervised Hebbian learning (strengthened connections when KC and MBON are active, weakened if KC is active without MBON; although note the conditions for weakening connections appear to differ from Huerta et al. 2004) is combined with supervised learning in which KC–MBON synapses are altered when reward/punishment occurs, with increase/decrease dependent on whether the respective MBON promotes a positive or negative response to the stimulus. In Cope et al. (2018), a related model is extended to include an accommodation process in KCs (reduced response to repetition of the same stimulus) and a feedforward collective inhibition signal from KCs to MBONs to account for the reported ability of bees to learn to distinguish “same” and “different” (in this case, repeat vs. novelty of successive stimuli) as an abstract concept (Giurfa et al. 2001). This model used an explicit three-factor rule in which simultaneous activation of KC and MBON is required for weight change and the sign of the change depends on the occurrence of reward or not.

The combination of new evidence that the KC–MBON STDP observed in locusts is gated or modulated by the activity of dopamine neurons (Cassenaer and Laurent 2012) with theoretical developments (Izhikevich 2007) motivated more explicit models of how a three-factor rule might operate in the MB. Izhikevich (2007) had proposed that a neuromodulatory reward signal was necessary to consolidate synaptic changes tagged by STDP. The MB circuit modeled in Wessnitzer et al. (2012) used only one MBON to which all KCs were connected and explicitly included a DAN assumed to respond to reinforcement. As in previous models, odor patterns produced sparse activation of the KC layer. KCs that spiked before the MBON (hence, likely produced the MBON response) had their synapse onto the MBON tagged with an exponentially decreasing signal. The subsequent occurrence of DAN activation would then strengthen KC synapses proportionally to the value of their tag.

This model, developed to explain olfactory conditioning paradigms (including nonelemental learning, in which the value of combinations of stimuli may differ in sign from the sum of their individual values), was also shown to be directly adaptable to a visual learning scenario that can account for route following in navigating insects (Ardin et al. 2016). This work built upon the hypothesis that navigating insects store memories of viewpoint-dependent vistas, and can subsequently use simple matching of the current visual input to these memories to determine a familiar view and hence a direction in which to proceed (Baddeley et al. 2012). Using views experienced along a route to the nest to produce sparse KC patterns, and a single MBON output as a familiarity signal, changing the weight of active KC synapses onto that MBON was shown to be sufficient to support navigation behavior. In practice, it was also shown that if a single output neuron is used, then the learning rule could be simplified to a binary change in connectivity of activated KCs to the MBON whenever a memory needs to be stored (e.g., signaled by DAN activity), effectively reducing this to the basic Kandelian mechanism described in the previous section.

Peng and Chittka (2017) use such a Kandelian form of the learning rule for KC–MBON connections in a bee-inspired MB model, but assume the existence of two DANS (for reward and punishment) that modulate exclusively the connections to one of two MBONs to independently encode appetitive and aversive memories. They also assume (in line with the emerging biological evidence mentioned above) that simultaneous KC–DAN activity results in synaptic depression rather than potentiation. The model is further extended by adding three-factor plasticity at the input-KC connections, which they argue is necessary to reproduce certain features seen in bee learning experiments, and, more generally, can enhance generalization. In Delahunt et al. (2018), a model of the olfactory lobe and MB calibrated to moth data uses a Hebbian learning rule for both input-KC and KC–MBON connections, gated by a reward signal indicated by widespread octopamine release.

Faghihi et al. (2017) also present a model in which dopamine modulates a (positive) weight change for coactive KC–MBON connections. They add a retrograde signal inspired by nitric oxide (NO) which is emitted by MBON activity and affects the coupling strength of input connections to KCs. In a simulated fly, they demonstrate this can support second-order conditioning.

## A return to prediction error

The previous models, although reflecting more closely some neurobiological details, in general did not include a number of increasingly salient features of the MB circuit. Some models (such as Peng and Chittka 2017) started to reflect the lobe and compartment structure by implementing separate signaling pathways for reward and punishment (DANs signaling different valence) linked to specific output units producing, respectively, aversion or attraction (MBONs of opposite valence). However, the full compartmental structure with multiple such pairings, each potentially with different learning properties (Aso et al. 2014; Hige et al. 2015; Aso and Rubin 2016; Takemura et al. 2017), had not been represented in models up to this point. Another crucial feature, also previously neglected in most models, is the extent of feedback connections from MBONs to DANs. Such feedback, allowing MBON activity to modulate the learning signal, had been anticipated in early discussions of the VUMmx1 neuron (Hammer and Menzel 1995; Hammer 1997) and in some early models (Smith et al. 2008) and was explicitly raised again as a potential substrate for PE in Terao et al. (2015) to account for blocking effects observed in cricket experiments. Several recent models have now explored this idea in more detail.

Eschbach et al. (2020) present original data for the feedback circuits in the larval brain, showing that DANs could receive >50% of their total dendritic input from direct or indirect feedback pathways from MBONs. Feedback motifs include within- and cross-compartment feedback, sometimes integrating inhibitory and excitatory inputs from compartments of opposing valence, and activation of feedback neurons is shown to be able to drive learning in a similar way to direct activation of DANs. A model was constructed by using the connectome data to define the model structure and then tuning it to perform several conditioning tasks. This used a plasticity rule based on the level of dopamine and activity of KC such that synapses are depressed if KC activity precedes DAN activity and increased if vice versa. The outcome produced a variety of DAN responses in the simulation, some, but not all, of which resembled PE (e.g., by showing a reduction in response when an expected reinforcer was omitted).

Bennett et al. (2021) explore what connection motifs exist (or might be predicted) in the KC–DAN–MBON system to support precise PE—that is, to ensure that (collective) MBON output accurately predicts the expected reinforcement and that (collective) DAN activity reflects the difference between this prediction and the reinforcement received. The compartmental structure is represented by two DANs (D+ and D−) that depress the KC connections to two MBONS (M− and M+) of opposite valence; the model also includes direct (fixed) KC–DAN connections and excitatory feedback from MBONs to their respective DANs. Thus DAN responses to KC patterns paired with reinforcement are reduced proportionally to the depression they have so far induced in the relevant KC–MBON synapses (negative feedback). They note that for this circuit to function, it requires an additional mechanism to potentiate weights (otherwise all KC–MBON synapses will be driven to 0) and introduce this as a parameter that will increase synaptic weights toward a constant, nonzero, value when KC is active but DAN activity is low. This model appears to capture well a range of existing experimental data for flies but predicts an upper bound to the magnitude of reward predictions that can be learned. They consequently develop an alternative model that can show unbounded predictive accuracy, which requires DANs that have bivalent responses (a base rate increased by reward and reduced by punishment, or vice versa) and both depressing and potentiating effects on KC–MBON synapses, as well as inhibitory feedback from MBONs to DANs of the same valence.

Springer and Nawrot (2021) provide a model that is more closely linked to specific *Drosophila* neuroanatomy but produces a similar (although less precise) effect of PE encoding in DANs because of MBON feedback. There is a similar use of two DANs to represent the PAM (reward) and PPL (punishment) clusters which, respectively, depress KC synaptic input to MBONs controlling avoidance or approach when KC and DAN activity coincide. In this case, four MBONs are used (corresponding to anatomically identified neurons in the *Drosophila* MB). Each DAN affects two MBONs, one of which controls behavior and also inhibits the activity of a neuron in the opposite valence pair; the other (the one receiving MBON inhibition) feeds back excitation to its own DAN. In effect, the initial learning signal from DANs includes both direct reinforcement and positive feedback from activated MBONs; as learning decreases the strength of KC input to the MBONs, the positive feedback, and hence the DAN learning signal, is reduced. As presented, there is no mechanism to increase the strength of synapses, and instead it is explored how this network can produce behavioral extinction of memory by generating a second memory of opposing valence when the reinforcer is omitted. Juergensen et al. (2024) present a similar model for larval learning, this time implemented in a spiking network, with only two MBONs that excite their own and inhibit their opposite DAN; a similar learning rule depresses KC–MBON weights for recently active KCs when a DAN is active, but in this case, depressed synapses are restored toward their initial state when MBONs spike.

Zhao et al. (2021) argue that simple associative learning in the form of a KC–MBON weight change proportional to a (temporal trace of) KC activity correlated with reinforcer strength cannot account for data in which different reinforcer strengths are delivered in different temporal relationships to odor (e.g., one large shock delivered at the start or end of odor presentation vs. multiple small shocks throughout). Instead, they propose an abstract learning rule that includes a PE term, subtracting the output from the reinforcer, which determines the degree and sign of synaptic change. Although not explicitly implemented as a computational model of a neural circuit, they suggest the rule could be realized in the MB either by a negative feedback connection from an MBON to its own DAN (reducing DAN activity and preventing further change) or by a “target-setting” influence of DANs on the KC–MBON connection (following a dendritic adaptation to somatic target mechanism suggested by Urbanczik and Senn 2014). They note that these two alternative implementations predict different effects of learning on DAN activity (respectively, decrease vs. increase), and of MBON silencing on learning (respectively, increase vs. decrease), but also note that contradictory evidence exists regarding these effects in *Drosophila* (Aso et al. 2014; Hige et al. 2015; Felsenberg et al. 2017), suggesting that perhaps both mechanisms coexist. The model is further extended to include subsystems for positive and negative valence and to allow weight change to be reversed by the experience of opposite valence.

## What lies beyond?

This recent focus on interpreting the dopamine signal in the MB as embodying PE learning is understandable, given that it would ground MB learning mechanisms in a strong theoretical foundation. Nevertheless, this is not the only possible interpretation of the feedback circuitry. Two recent models take contrasting approaches to explore further how circuit properties that have been uncovered in recent connectomic and related work might be functionally understood.

In Gkanias et al. (2022), a partial MB circuit (comprised of six DANs and six MBONs, plus KCs to carry the input pattern) is constructed “bottom-up” by linking together evidence from several well-explored microcircuits in *Drosophila* (Cohn et al. 2015; Ichinose et al. 2015; Felsenberg et al. 2018; Pavlowsky et al. 2018; Li et al. 2020; McCurdy et al. 2021). On the basis of symmetry (for learning attractive vs. aversive events) some additional neurons and connections are identified resulting in a tightly linked architecture that supports both rapid learning and transfer to long-term memory. The circuit uses multiple connections between MBONs and from MBONs to DANs, but notably, none of these form a direct negative feedback loop as required for PE. Instead, positive feedback loops help to stabilize learning processes and transfer information between compartments. This model also departs from most previous work in specifying a learning rule, inspired by Handler et al. (2019), that alters (in different ways) the synapses onto MBONs of both active and inactive KCs when DANs are active (consistent also with biological results from Berry et al. 2018, Faghihi et al. 2017, and Schleyer et al. 2018, 2020). The model outputs are first compared directly to calcium imaging data from the respective neurons and then tested in a simulated agent. Interestingly, the latter reveals asymmetric learning indices for punishment versus reward, despite the underlying circuit being entirely symmetric.

Jiang and Litwin-Kumar (2021) take an alternative “bottom-up” approach by using some architectural features of the MB to constrain a recurrent network (consisting of 200 KCs, 20 MBONs, 20 DANs, and 60 feedback neurons) which is then parameterized through stochastic gradient descent to produce outputs matching those expected for various learning paradigms. Specifically, KCs activate MBONs, each DAN acts (in a compartmentalized fashion) to modify KC connections for one MBON, feedback neurons get inputs from reinforcement or contextual states, and all MBONs and all feedback neurons can potentially connect to one another and to DANs. The KC–MBON plasticity is governed by the relative timing of activation of the relevant DAN and KC, again following the phenomenology of synaptic change described in Handler et al. (2019); the model is also later enhanced to perform more continuous learning by adding a potentiation term for all synapses when a DAN is active, consistent with observations from Cohn et al. (2015). Interesting observations from the optimization process include that simple conditioning does not require recurrent connectivity, but extinction and second-order conditioning do, and that adding KC–DAN connections does not seem to provide any additional function. The emerging DAN response properties are highly varied, including encoding the valence of the US, the predicted valences of trained CS, showing reduced response to omission of an expected reward, or increased response to omission of reward of opposite valence. The collective dynamics of the DANs can be interpreted as encoding PE, but the diversity of responses appears to be important for function. In addition, optimizing for a different target behavior such as novelty detection produced different dominant DAN dynamics. Using this circuit to control an agent moving in a simulated odor gradient revealed a continuing role for DAN-modulated plasticity during the approach to odors and an additional correlation of DAN activity with movement.

In summary, neither of these “bottom-up” models assumed that PE played a fundamental role and both concluded that a richer interpretation of the function of MBON feedback to DANs is warranted. Looking forward, it will be relevant to explore further in computational models the potential role of other forms of plasticity in the MB than just the KC–MBON synapse that has been the focus of this review. The potential role of plasticity in the calyx (i.e., changing how inputs map to KCs [Peng and Chittka 2017] or adaptive responses of KCs to input [Cope et al. 2018]) has already been briefly mentioned. But it also seems plausible that KC–DAN, MBON–MBON, and MBON–DAN connections could undergo modulation or long-term change. Consideration of the MB connectome for larval and adult *Drosophila* (Eichler et al. 2017; Li et al. 2020) has highlighted extensive KC–KC connectivity. Some earlier MB-inspired models suggested that such connectivity could allow dynamic recurrent patterns to emerge in KC firing (Payne et al. 2010; Arena et al. 2013), followed by adaptive linear readout by the MBONs, resembling a reservoir network (Schrauwen et al. 2007). A more recent model has explored if adaptive connections between KCs could contribute to learning sequences of sensory patterns in a visual navigation context (Zhu et al. 2023).

Another consideration for future work is the need to strengthen our understanding and intuitions about how the surrounding context of this circuitry may determine its function. This includes the rest of the brain, the body, and the environment of a learning animal. A more ecological consideration of what associative learning is for may be important. For example, few experimental paradigms, or the simulations that mimic them, represent the natural conditions in which animals appear able to extract relevant regularities and make continuous control decisions when experiencing a complex and continually varying sensory stream. A more comparative approach, understanding how the form and function of the MB differ across species, is perhaps also overdue.

In all neural modeling, there is an ongoing tension between models that explore the rich variety of the circuit, and those that focus on unifying simplifications, with the hope of more fundamental understanding. In particular, the possibility of finding a direct mapping from MB properties to effective, general learning algorithms seems attractive. However, a risk is that interpreting the MB through the lens of existing theory, such as PE learning, might distort our perspective. Although the plurality of existing models, and their apparent failure to converge, may seem disappointing, a more positive perspective is that the computation and capabilities of the MB are still wide open questions, and we can look forward to the insights another 25 years of research will bring.
